# TET2 downregulation enhances the antitumor efficacy of CD19 CAR T cells in a preclinical model

**DOI:** 10.1186/s40164-025-00609-8

**Published:** 2025-02-26

**Authors:** Yeongrin Kim, Moonjung Jeun, Heung Kyoung Lee, Ji U Choi, Simon Park, Chi Hoon Park

**Affiliations:** 1 Data Convergence Drug Research Center, Korea Research Institute of Chemical Technology, Daejeon, 34114 Republic of Korea; 2 College of Pharmacy, Chungnam National University, Daejeon, Republic of Korea; 3 Medicinal Chemistry and Pharmacology, Korea University of Science and Technology, Daejeon, Republic of Korea; 4 Abtironbio, Hanam-Si, Republic of Korea

**Keywords:** CAR T cell, TET2, shRNA, Cancer, Immunotherapy, CAR T cell therapy

## Abstract

Chimeric antigen receptor (CAR) T cell therapy has demonstrated significant clinical efficacy in patients with hematologic cancers. However, long-term follow-up studies indicate that only 50% of patients remain in complete remission after three years. To overcome these limitations, we investigated a strategy to enhance the antitumor activity of CAR T cells through gene modification. Based on previous research results demonstrating that CAR T cells with disrupted TET2, a methylcytosine dioxygenase, exhibit enhanced antitumor effects compared to conventional CAR T, we developed CAR T cells in which TET2 is downregulated by TET2 shRNA. Among the screened TET2-specific shRNAs, TET2-shRNA-1 was identified as the most effective sequence for gene silencing. Using this sequence, we constructed an all-in-one vector co-expressing CD19 CAR and TET2 shRNA. In vitro studies demonstrated that TET2 knockdown CD19 CAR T cells exhibited comparable cytolytic activity against CD19-positive cancer cells compared to conventional CD19 CAR T cells. However, interestingly, in xenograft mouse model using NSG mice, TET2 knockdown CAR T cells showed significantly improved antitumor activity compared to conventional CAR T cells. Our study demonstrates that shRNA-mediated knockdown of TET2 is a promising strategy to enhance the antitumor activity of CD19 CAR T cells in a preclinical model.

**Supplementary Information:**

The online version contains supplementary material available at 10.1186/s40164-025-00609-8.

**To the editor**

Chimeric antigen receptor (CAR) T cells have demonstrated remarkable clinical efficacy in patients with hematologic malignancies [1]. However, long-term follow-up studies of cluster of differentiation 19 (CD19) CAR T cell therapy indicate that only 50% of the patients remained in complete remission over 3 years [2]. To address these challenges, various studies are being conducted to improve the anticancer effects of CAR T cells [3–7]. Intriguingly, Fraietta et al. reported that unintentional disruption of the Ten-Eleven Translocation-2 (TET2) gene due to CAR gene insertion markedly enhanced the efficacy of CD19 CAR T cells in a chronic lymphocytic leukemia (CLL) patient [8]. TET2 encodes methylcytosine dioxygenase that mediate the DNA demethylation [9, 10]. In this study, we aimed to enhance the antitumor activity of CD19 CAR T cells through short hairpin RNA (shRNA)-mediated TET2 silencing.

First, we designed an all-in-one vector co-expressing CD19 CAR and TET2 shRNA (Fig. 1A). The sequence of TET2-shRNA-1 was adopted from published paper (Fig. S1A) [11], and sequences for TET2-shRNA-2 to 7 were obtained using BLOCK-iT™ RNAi Designer tools (Thermofisher). Each shRNA was transduced into 293 T cells by lentiviral vector. As a results, TET2-shRNA-1, 4, 6 and 7 reduced the expression of TET2. Among them, TET2-shRNA-1 reduced the expression of TET2 most effectively (Fig. 1B and Fig. S1B).

**Fig. 1:**
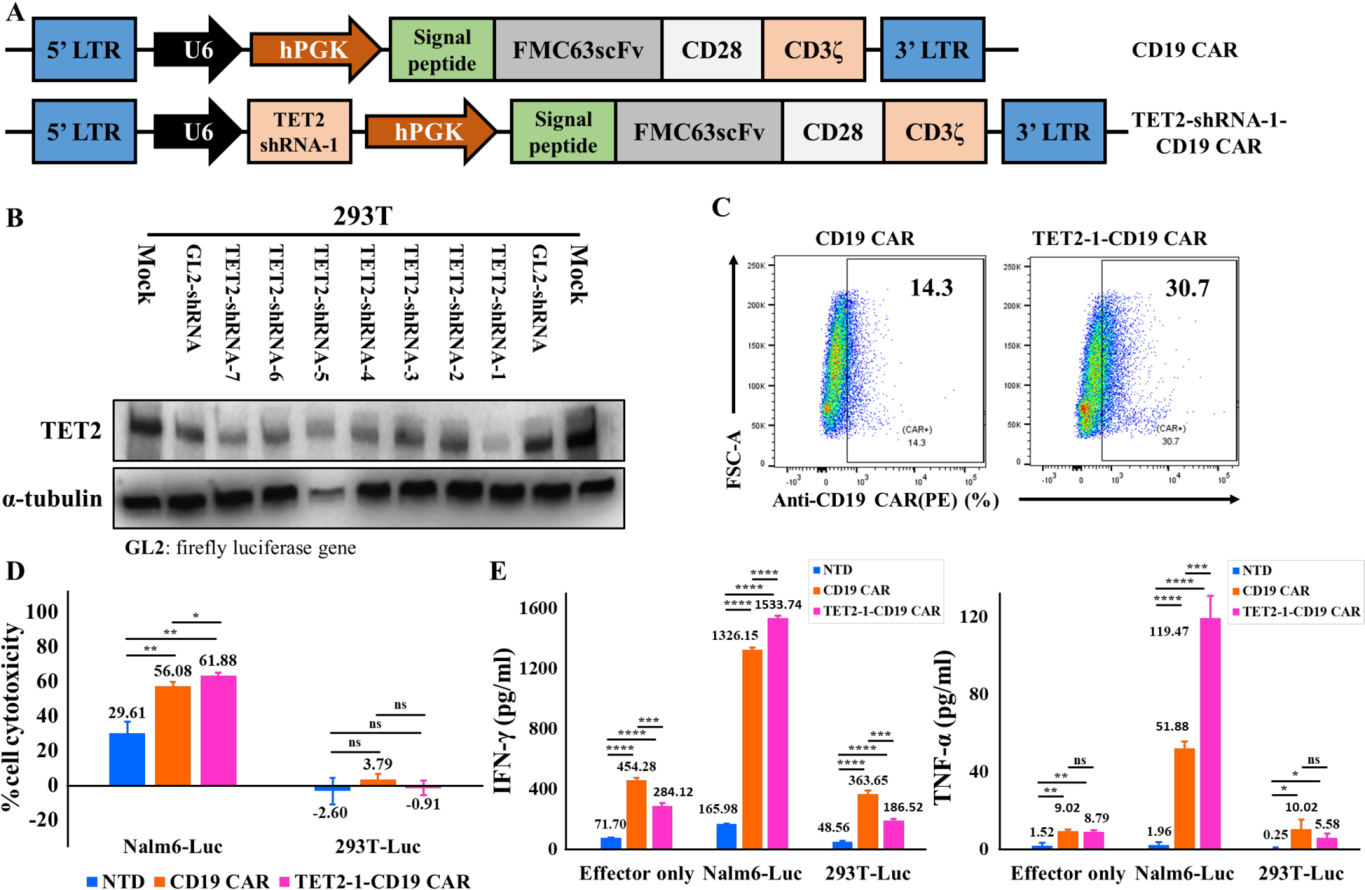
CAR-dependent cytotoxic function or cytokine secretion of TET2-1-CD19 CAR T cells in vitro. **A** Schematic of CD29 CAR and TET2-1-shRNA-CD19 CAR constructs. **B** Western blot analysis of TET2. Total proteins in the samples were separated using SDS-PAGE. α-tubulin bands represent loading controls. GL2-shRNA is a negative control. **C** CD19 CAR expression on CD19 CAR T cells or TET2-1-CD19 CAR T cells. **D** Percent cytotoxicity of NTD, CD19 CAR, or TET2-1-CD19 CAR T cells against CD19-positive Nalm6-Luc or negative 293 T-Luc cells. Effector cells (1 × 10^5^) and target cells (1 × 10^4^) were co-cultured for 4 h. **E** Nalm6-Luc or 293 T-Luc cells were co-incubated with NTD, CD19 CAR or TET2-1-CD19 CAR T cells at an E:T ratio of 3:1 for 24 h. The IFN-γ or TNF-α concentration in supernatants was measured by ELISA. Statistical significance was evaluated using the unpaired t-test, (n = 3, mean $\pm$ SD), (ns: non-significant, *p < 0.05, **p < 0.01, ***p < 0.001; ****p < 0.0001), (Two-tailed p value)

Next, we demonstrated anticancer efficacy of CD19 CAR T cells in which TET2 is downregulated by TET2-shRNA-1 (TET2-1-CD19 CAR T) in vitro. We evaluated cytolytic activity of CD19 CAR T cells or TET2-1-CD19 CAR T cells (Fig. 1C and Fig. S2) against CD19-positive Nalm6 expressing firefly luciferase (Nalm6-Luc) cells. TET2-1-CD19 CAR T cells exhibited undiminished cytotoxicity against Nalm6-Luc cells compared to CD19 CAR T cells (Fig. 1D). These results were consistently observed across a repeated experiment (Fig. S3A). Subsequently, we conducted lysis assay to compare anticancer activity between CD19 CAR T and TET2-1-CD19 CAR T cells against additional CD19-positive Daudi expressing firefly luciferase (Daudi-Luc) cells. TET2-1-CD19 CAR T cells exhibited comparable cytotoxicity against Daudi-Luc cells compared to CD19 CAR T cells also (Fig. S3B). To assess cytokine secretion function of CAR T cells, we measured concentrations of various cytokines secreted by effector T cells. Upon recognition of the CD19 antigen, TET2-1-CD19 CAR T cells secreted IFN-γ, TNF-α, GM-CSF, IL-2 and Granzyme B at concentrations comparable to those of CD19 CAR T cells (Fig. 1E and Fig. S4). In addition, TET2 knockdown did not noticeably alter the expression level of phenotypic (CD4 and CD8) and exhaustion (PD-1, Tim-3 and LAG-3) markers on CAR T cells (Fig. S5 and S6). These results demonstrated that TET2 knockdown does not adversely affect CAR-dependent cytotoxic function or cytokine secretion of CD19 CAR T cells in vitro.

Finally, we evaluated the antitumor activity of TET2-1-CD19-CAR T cells in xenograft mouse model. We conducted independent experiments with effector T cells from different donors. All NOD.Cg-PrkdcscidIl2rgtm1Wjl/SzJ (NSG) mice were intravenously injected with Nalm6-Luc cells, and divided into four groups. The following day, each group was intravenously administered PBS, NTD, CD19 CAR, or TET2-1-CD19 CAR T cells (Fig. 2A and Fig. S7A). TET2-1-CD19 CAR T cells significantly improved the survival rate of mice compared to other groups (Fig. 2B, C, S7B and S7C). As shown in Fig. 2D and Supplementary Figure S7D, the luminescence observed in mice was dramatically reduced in the TET2-1-CD19 CAR T group. These results demonstrate that TET2-1-CD19 CAR T cells effectively reduce tumor burden and significantly increase the survival rate of mice.

**Fig. 2:**
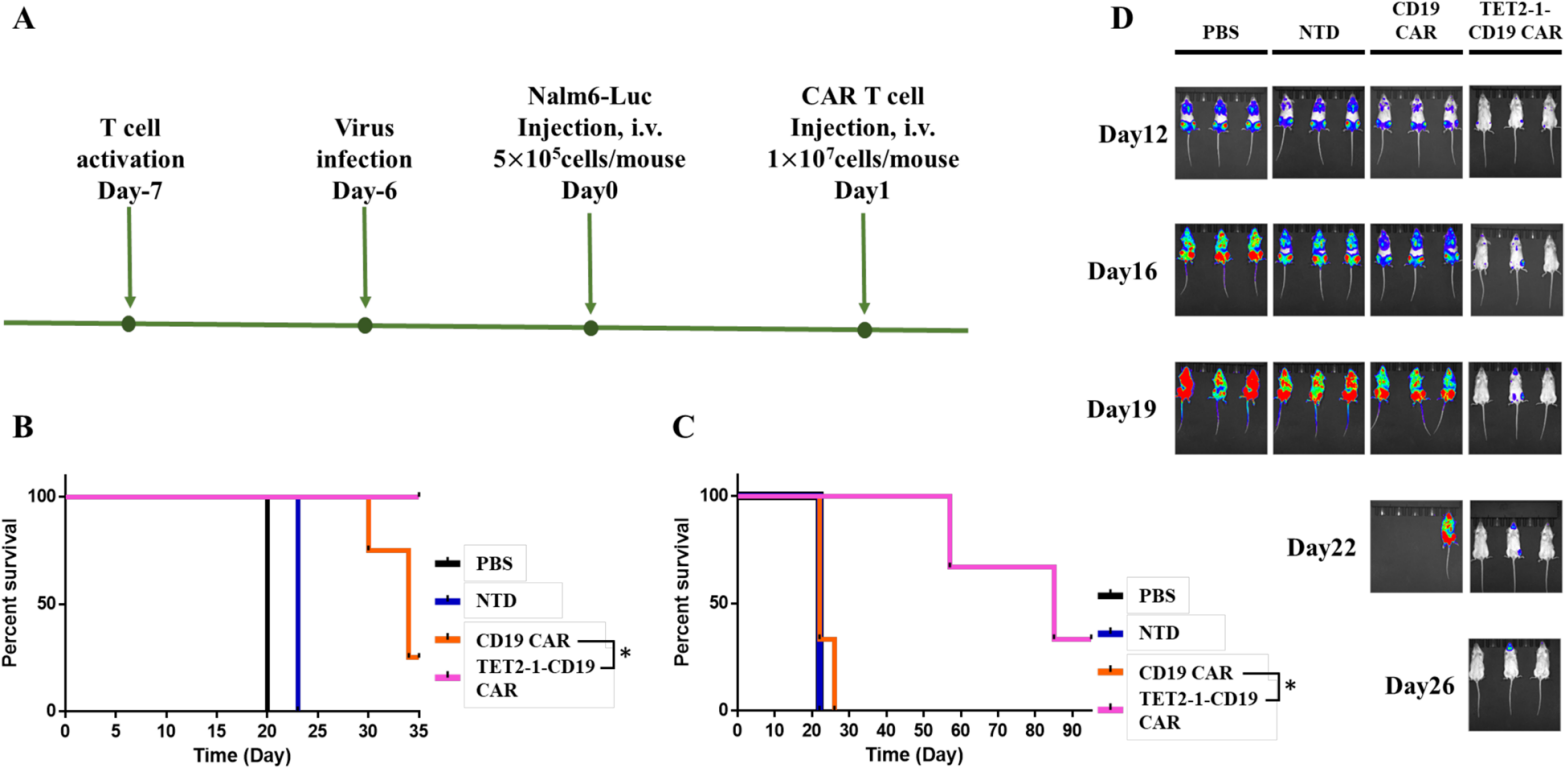
The antitumor efficacy of TET2-1-CD19 CAR T cells in a xenograft mouse model. **A** Schematic overview of in vivo study. **B**, **C** Kaplan–Meier survival curves of NSG mice treated with PBS, NTD, CD19-CAR, or TET2-1-CD19-CAR T cells one day after injection of Nalm6-Luc cells. The survival rates of mice were monitored up to Day 35 (**B**) or Day 95 (**C**). **B** Four mice were used in the CD19 CAR and TET2-1-CD19 CAR groups. **C** Three mice were used per group. **D** Bioluminescence image of the mice used in Fig. 2(**C**). Survival analyses were compared using the log-rank Mantel-Cox test (*p < 0.05)

In this study, we demonstrate that shRNA-mediated TET2 downregulation significantly enhance the antitumor activity of CAR T cells in a preclinical model. However, shRNA-mediated TET2 knockdown, as an epigenetic programming approach, may have potential risks such as unchecked proliferation of CAR T cells, similar to those observed with CRISPR-Cas9-mediated TET2 knockout [12]. However, shRNA-mediated knockdown downregulates mRNA, not gene. Therefore, shRNA-mediated knockdown could be used as an alternative epigenetic programming method to CRISPR-Cas9-mediated TET2 knockout.

In conclusion, we found that TET2-downregulated CAR T cells dramatically improved the CAR T cell efficacy in a preclinical model. We suggest that shRNA-mediated TET2 knockdown strategy could be considered as an alternative option for immunotherapy.

## Supplementary Information

Supplementary Material 1.

## Data Availability

No datasets were generated or analysed during the current study.

## Abbreviations

CAR: Chimeric antigen receptor
CD19: Cluster of differentiation 19
TET2: Ten-eleven translocation-2
CLL: Chronic lymphocytic leukemia
shRNA: Short hairpin RNA
NSG: NOD.Cg-PrkdcscidIl2rgtm1Wjl/SzJ
