# Supplementary material for: TET2 downregulation enhances the antitumor efficacy of CD19 CAR T cells in a preclinical model
Source: Exp Hematol Oncol. 2025 Feb 26;14:23. doi: 10.1186/s40164-025-00609-8 (PMC11866829; doi:10.1186/s40164-025-00609-8)
Supplement: Supplementary file 1 — Supplementary Material 1. [file 40164_2025_609_MOESM1_ESM.docx]

**TET2 downregulation enhances the antitumor efficacy of CD19 CAR T cells in a preclinical model**

**Supplementary Materials and Methods:**

**Cells and cell culture**

Nalm6 cells were purchased from Leibniz-Institute DSMZ-German Collection of Microorganisms and Cell Cultures (DSMZ) (Germany). 293T cells were purchased from (American Type Culture Collection, Manassas, VA, USA). Nalm6 cells were cultured in RPMI-1640 (Hyclone, Logan, UT, USA, Cat#SH30027.01) containing 10% fetal bovine serum (FBS) (Thermo Fisher, Waltham, MA, USA, Cat#16000-044). 293T cells were maintained in DMEM (Hyclone, Cat#SH30243.01) supplemented with 10% FBS. Nalm6-Luc and 293T-Luc cells were transduced with firefly luciferase gene to express luminescence for cell cytotoxicity assay.

**CAR constructs**

All CAR constructs comprised a scFv extracellular domain, a CD28 transmembrane domain, and a CD3ζ intracellular domain. We used FMC63 targeting CD19 as the scFv extracellular domain and prepared FMC63-CD28-CD3ζ CAR and TET2-shRNA-1-FMC63-CD28-CD3ζ CAR constructs through gene cloning. The CAR gene and TET2-shRNA-1 were inserted into all-in-one lentivirus vector, in which hPGK promoter expresses CAR gene and U6 promoter transcribes TET2-shRNA-1.

**PBMC isolation**

Peripheral blood mononuclear cells (PBMC) were isolated from whole blood obtained from the Korean Red Cross Blood Services. Human whole blood was also obtained from healthy donors who provided written informed consent, according to protocols approved by the Korea National Institute for Bioethics Policy Institutional Review Board (approval no. P01-201607-31-003). All experimental protocols involving peripheral PBMCs were approved by the Institutional Review Board. PBMCs were isolated as previously described [1].

**CAR lentivirus production**

Packaging, envelopes, and transfer vectors were prepared for lentivirus production. The packaging vector is psPAX2 (Addgene, Watertown, MA, USA, Cat#12260), the envelope vector is pMD2.G (Addgene, Cat#12259), and the transfer vector is all-in-one vector. The all-in-one vector is based on pLKO.1 (Addgene, Cat#8453). The all-in-one vector includes the U6 promoter and the hPGK promoter, where the U6 promoter drives the expression of shRNA, and the hPGK promoter drives the expression of the CD19 CAR. The transfer vector was co-transfected into 293T cells along with the packaging and envelope vectors. After 24 h, the culture medium was replaced with fresh complete DMEM. The culture media were harvested at 48 and 72 h, and filtrated using a 0.45 μm PES membrane filter (Merck Millipore, Burlington, MA, USA, Cat#SLHP033RB).

**T cell activation and CAR T cell production**

Cryopreserved PBMCs were rapidly thawed in a 37 ℃ water bath. To selectively activate T cells within the PBMCs population, human T-activator CD3/CD28 Dynabeads (Thermo Fisher, Cat#11132D) were added at a bead-to-cell ratio of 1:5. After 24 h, the T cells were transduced with the CAR lentivirus containing 8 μg/ml polybrene (Sigma-Aldrich, St. Louis, MO, USA, Cat#H9268-5G) by spinoculation at 1,500 × g for 90 min at 25 ℃. T cells were cultured in RPMI-1640 medium supplemented with 10% FBS and 200 IU/ml human recombinant IL-2 (R&D Systems, Minneapolis, MN, USA, Cat#202-IL).

**Western blot analysis**

Cells were centrifuged at 1,500 × g for 3 min. The cell culture medium was replaced with 1$\times$ sample buffer (10% glycerol, 2% SDS, 50 mM Tris (pH 6.8), 3% β-mercaptoethanol, and 0.02% bromophenol blue). Cell lysates were boiled at 100 ℃ for 10 min. Samples were separated by gel electrophoresis on a 4–15% precast polyacrylamide gel (Bio-Rad, Hercules, CA, USA, Cat#456-1086) and transferred onto a PVDF membrane (Bio-Rad, Cat#1620177). Blots were blocked with 1$\times$TBST with 2% Tween20 (400 mM Tris, 3 M Sodium chloride, 2% Tween 20) containing 5% skimmed milk (LPS solution, South Korea, Cat#SKI500) for 90 min at 25 ℃ and washed three times with 1$\times$TBST with 2% Tween 20. Blots were incubated with primary antibodies overnight at 4 ℃. TET2 expression was verified using the TET2 (D6B9Y) antibody (Cell Signaling Technology, Danvers, MA, USA, Cat#18950). The primary antibody-treated blots were incubated with horseradish peroxidase (HRP)-tagged secondary antibodies for 90 min at 25 ℃. For detection of separated proteins, blots were treated with the ECL reagent (Thermo Fisher Scientific, Cat#32209). In this study, α-Tubulin antibody (Cell Signaling Technology, Cat#2144) or β-actin antibody (Cell Signaling Technology, Cat#4967) were used as loading control. Images were analyzed using the Sensi-Q2000 chemidoc system (LugenSci, South Korea, Cat#LGW-2000) and the Sensi-Q2000 software.

**Cytotoxicity assays**

To test the cytolytic activity of CAR T cells, cytotoxicity assays were performed using the Bright-Glo luciferase assay system (Promega, Fitchburg, WI, USA, Cat#E2650) according to the manufacturer’s protocols. The activated T cells were transduced with the CD19 CAR or TET2-shRNA-CD19 CAR lentivirus. CAR T cells were co-cultured with target cells expressing luciferase at an E:T ratio of 10:1 for 4 h or 24 h in a 96-well cell culture plate. After co-incubation, Bright-Glo solution was added to the same volume of the cell mixture in the 96-well plates. The plates were shaken for 5 min at 25 ℃, followed by the transfer of the mixture into white flat-bottomed 96-well plates (Costar, Glendale, AZ, USA, Cat#3917). The luminescence signals were detected using an EnSpire alpha reader (PerkinElmer, Shelton, CT, USA). The cytotoxicity was calculated using the following formula: (1-(luminescence [target cells with effector cells]) / (luminescence [target cells only])) ×100.

**Flow cytometry**

To detect CAR expression on CAR T cells, non-transduced T cells or CAR T cells (1 × 10^6^ cells) were suspended in 100 μl of cold stain buffer (PBS 0.2% BSA, 0.08% sodium azide) and stained with 2 μl of Fc-tagged human CD19 protein (ACROBiosystems, Newark, DE, USA, Cat#CD9-H5251) for 20 min on ice. After washing with the cold stain buffer, 2 μl of PE anti-human IgG Fc Antibody (BioLegend, San Diego, CA , USA, Cat#410707) was added to the 100 μl cells and incubated on ice for 20 min. The cells were rinsed with the cold stain buffer and fixed for 10 min at 4 °C using 4% Paraformaldehyde solution (Biosesang, South Korea, Cat#PC2031-050-00). After washing the cells, the cells were prepared in a 5 ml of cell-strainer cap polystyrene tube (Corning, Burlington, MA, USA, Cat#352235). Additionally, FITC anti-human CD3 Antibody (BioLegend, Cat#300406), APC/Cyanine7 anti-human CD4 Antibody (BioLegend, Cat#300518), Pacific blue anti-human CD8 Antibody (BioLegend, Cat#344718), PE anti-human PD-1 Antibody (BioLegend, Cat#329906), PE anti-human Tim-3 Antibody (R&D Systems, Cat#FAB2365P), or APC anti-human LAG-3 Antibody (BioLegend, Cat#369212) were used to detect CD3, CD4, CD8, PD-1, Tim-3, or LAG-3 on T cells, respectively. Flow cytometry was performed on a BD FACSCanto II (BD Biosciences, Franklin Lakes, NJ, USA) and data were analyzed with FlowJo (Treestar, Ashland, OR, USA).

**Measurement of cytokine concentrations**

Effector cells (3 × 10^4^ cells) were co-cultured with target cells (1 × 10^4^) for 24 hours. The cell supernatant was harvested by using centrifugation at 13,000 rpm for 10 minutes, and the secreted cytokine concentration was evaluated using ELISA kits (Biolegend, IL-2 Cat#431804, GM-CSF Cat#432004, Granzyme B Cat#439204, TNF-α Cat#430204, IFN-γ Cat#430104).

**In vivo studies**

All the mouse experiments were performed in accordance with the guidelines approved by the Laboratory Animal Care and Use Committee of the Korea Research Institute of Chemical Technology. Female NSG (NOD.Cg-PrkdcscidIl2rgtm1Wjl/SzJ) mice used in this study were purchased from Jackson Laboratory (Japan). NSG mice were engrafted with 5 × 10^5^ Nalm6-Luc cells via tail vein injection. One day later, 1 × 10^7^ NTD, CD19 CAR, TET2-1-CD19 CAR T cells were injected intravenously. The investigator was blinded to the group allocation. Survival curves were plotted using the Kaplan–Meier method. For bioluminescent imaging in vivo, mice were intraperitoneally injected with 150 mpk/mouse XenoLight D-Luciferin Potassium Salt (PerkinElmer, Cat# K9921PE).

**Statistical analysis**

Statistical significance was assessed using the unpaired t-test (mean ± SEM) (ns: non-significant, *p < 0.05, **p < 0.01, ***p < 0.001, ****p < 0.0001; Two-tailed p value). Survival analysis was performed using the log-rank Mantel-Cox test (ns: non-significant, *p < 0.05, **p < 0.01, ***p < 0.001, ****p < 0.0001). Data analysis was conducted using GraphPad Prism version 6 (GraphPad Software, La Jolla, CA, USA).

**References**

[1] Kim, Y., Lee, D.Y., Choi, J.U. et al. Optimized conditions for gene transduction into primary immune cells using viral vectors. Sci Rep 13, 12365 (2023). https://doi.org/10.1038/s41598-023-39597-2

**Supplementary Figures and Figure Legends:**


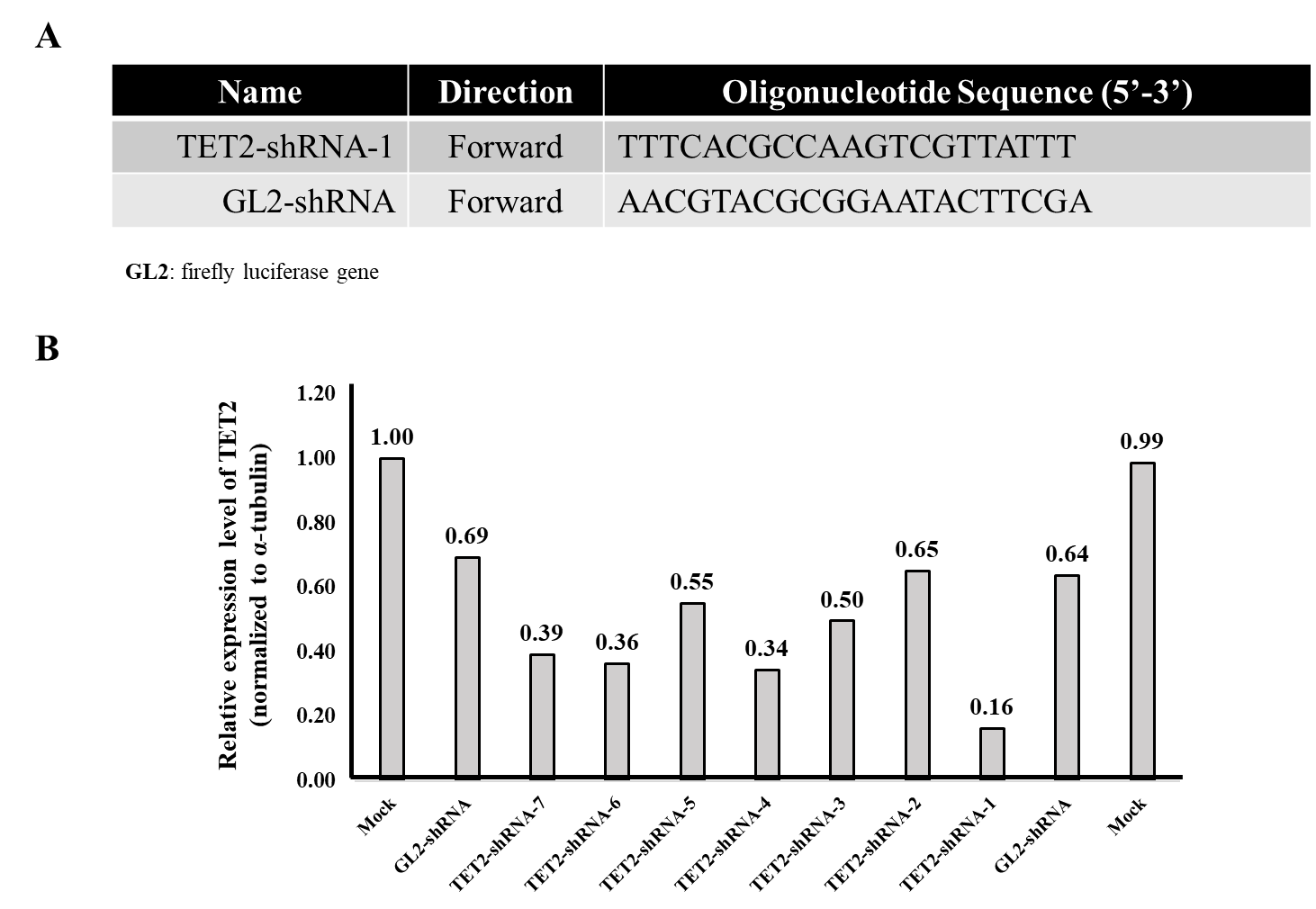


**Fig. S1** shRNA-mediated downregulation of TET2. (**A**) Sequences of TET2-shRNA-1 and GL2-shRNA. (**B**) Relative expression level of TET2 proteins (normalized to α-tubulin).


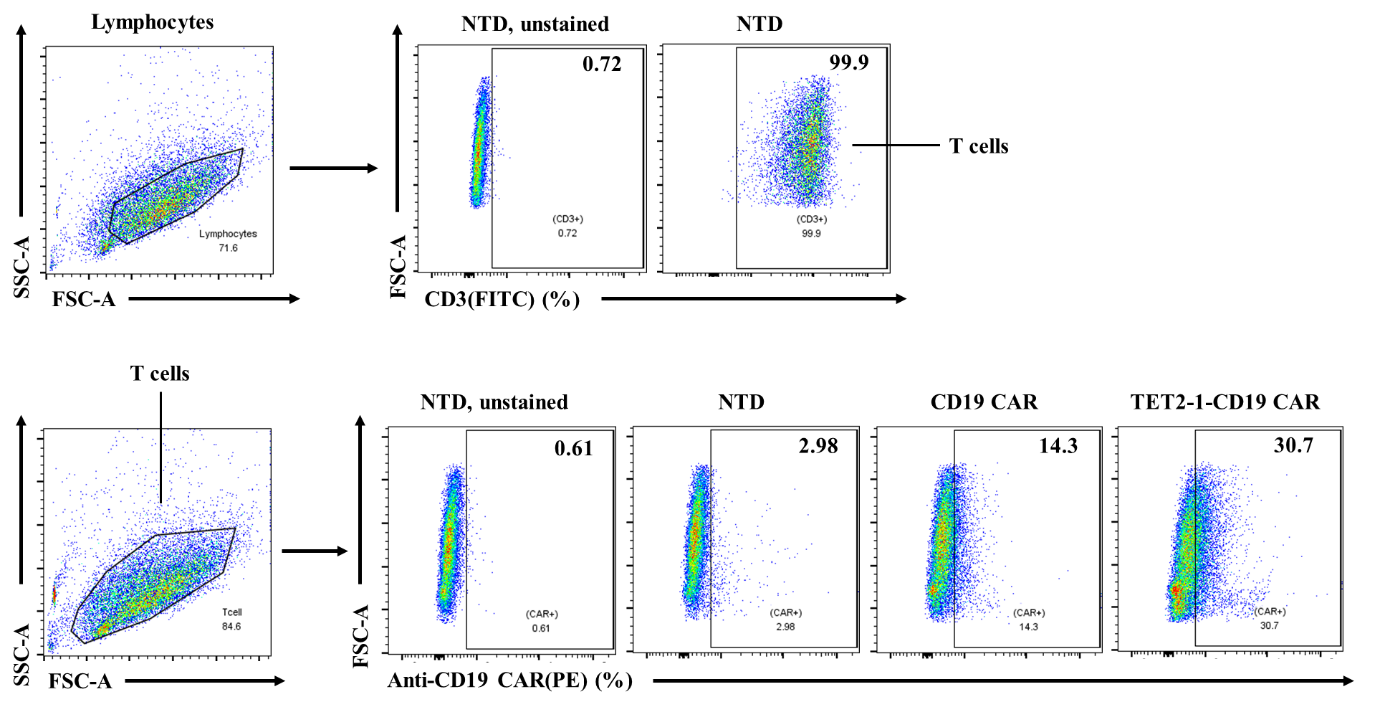


**Fig. S2** The CD3 expression on T cells and CD19 CAR expression on CAR T cells or TET2-1-CD19 CAR T cells. The expression level of CD3 and CD19 CAR were analyzed by flow cytometry.


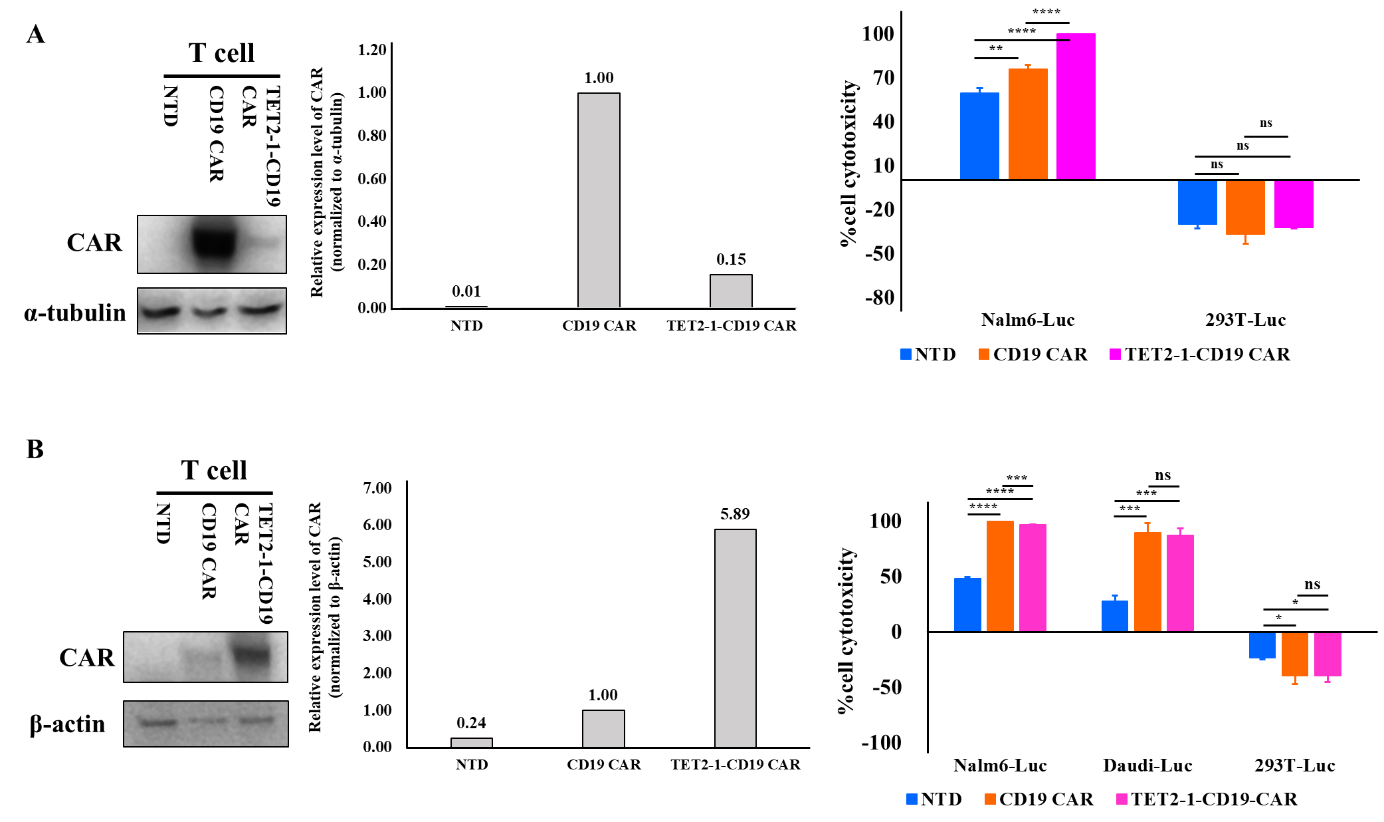


**Fig. S3** The anticancer activity of CD19 CAR T cells or TET2-1-CD19 CAR T cells in vitro. (**A**) Western blot analysis of CD19 CAR and relative expression level of CD19-CAR (normalized to α-tubulin). Total proteins in the samples were separated using SDS-PAGE. α-tubulin bands represent loading controls. Percent cytotoxicity of NTD, CD19 CAR T or TET2-1-CD19 CAR T cells against CD19-positive Nalm6-Luc or CD19-negative 293T-Luc cells. Effector T cells (1×10^5^) and target cells (1×10^4^) were co-cultured for 24 h. (**B**) Western blot analysis of CD19 CAR and relative expression level of CD19-CAR (normalized to β-actin). Total proteins in the samples were separated using SDS-PAGE. β-actin bands represent loading controls. Percent cytotoxicity of NTD, CD19 CAR T or TET2-1-CD19 CAR T cells against CD19-positive Nalm6-Luc, Daudi-Luc or CD19-negative 293T-Luc cells. Effector T cells (1×10^5^) and target cells (1×10^4^) were co-cultured for 24 h. Statistical significance was evaluated using the unpaired t-test, (n=3, mean$\pm$SD), (ns: non-significant, *p < 0.05, **p < 0.01, ***p < 0.001; ****p < 0.0001), (Two-tailed p value).


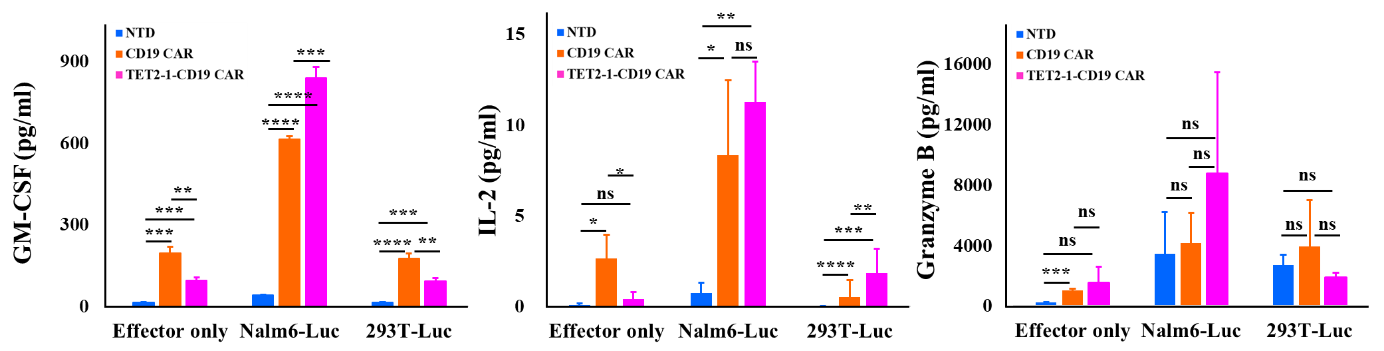


**Fig. S4** The CAR-dependent cytokine secretion of NTD, CD19 CAR or TET2-1-CD19 CAR T cells. Nalm6-Luc or 293T cells were co-incubated with NTD, CD19 CAR or TET2-1-CD19 CAR T cells at an E:T ratio of 3:1 for 24 h. GM-CSF, IL-2 or Granzyme B concentration in supernatants was measured by ELISA. Statistical significance was evaluated using the unpaired t-test, (n=3, mean$\pm$SD), (ns: non-significant, *p < 0.05, **p < 0.01, ***p < 0.001; ****p < 0.0001), (Two-tailed p value).


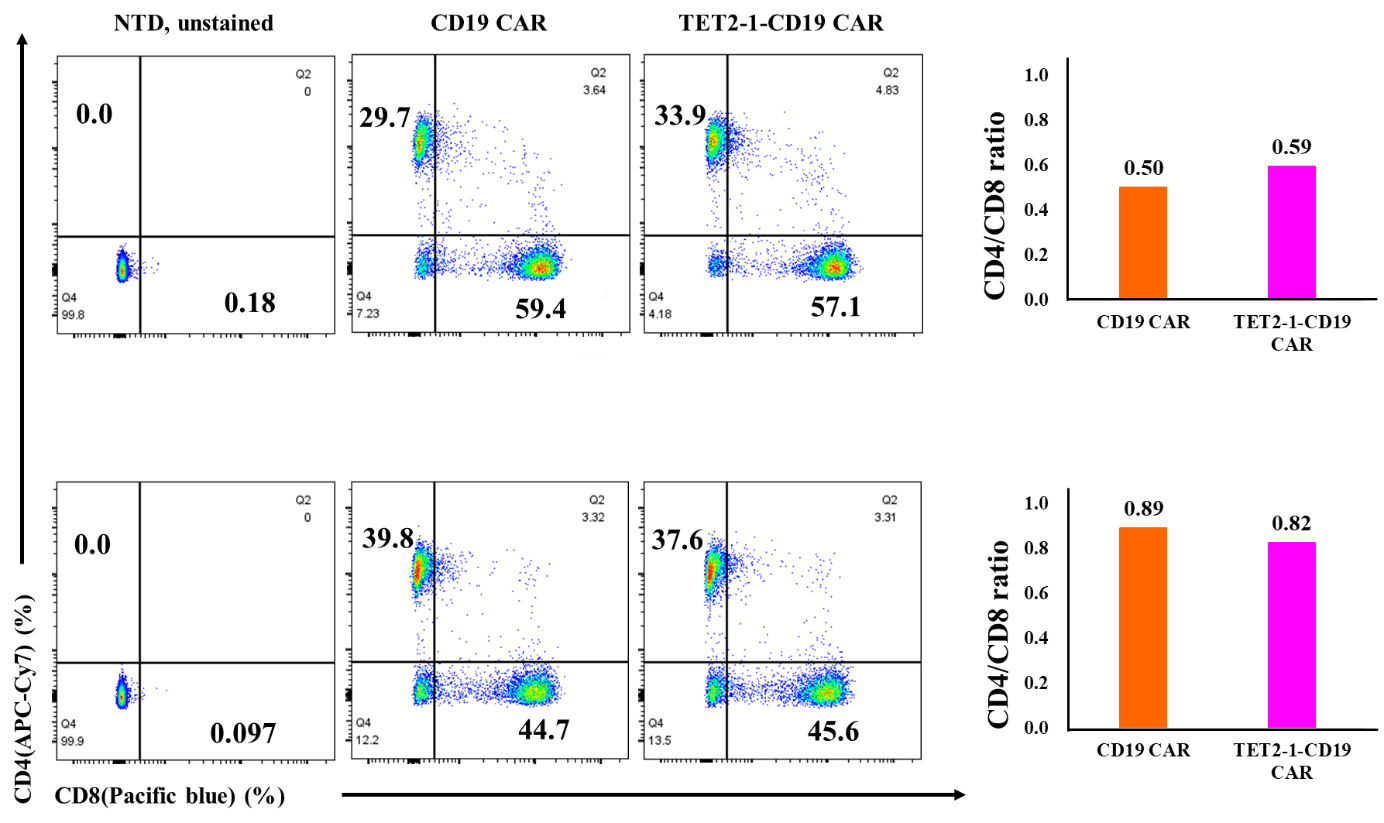


**Fig. S5** The expression level of phenotypic markers (CD4 and CD8) on CD19 CAR T cells or TET2-1-CD19 CAR T cells from different donors. The expression levels of CD4 and CD8 were analyzed by flow cytometry. The CD4/CD8 ratio was calculated for each donor using the results of flow cytometry analysis.

\


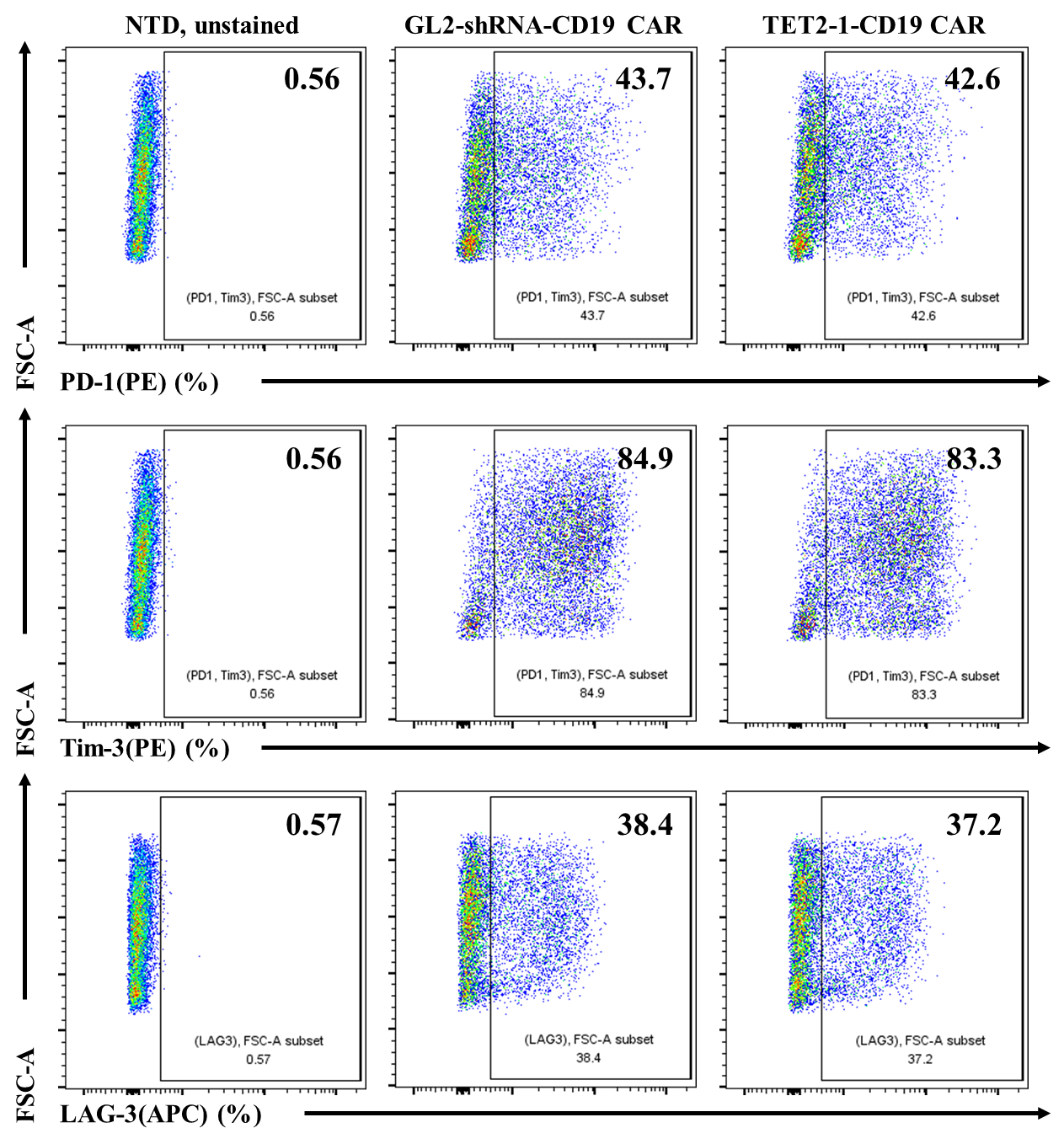


**Fig. S6** The expression level of exhaustion markers (PD-1, Tim-3 and LAG-3) on GL2-shRNA-CD19 CAR T cells or TET2-1-CD19 CAR T cells. The expression levels of PD-1, Tim-3 and LAG-3 were analyzed by flow cytometry.


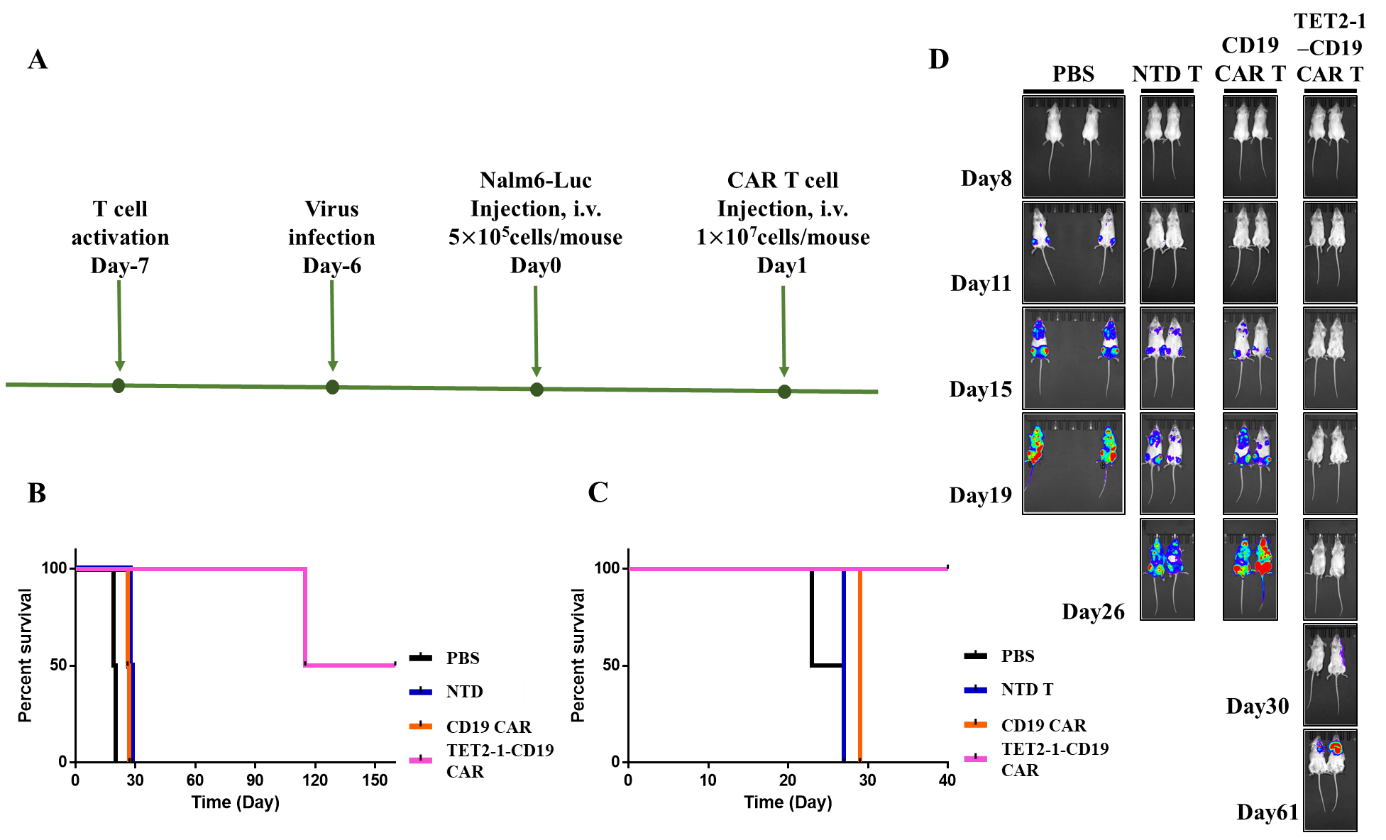


**Fig. S7** The antitumor efficacy of TET2-1-CD19 CAR T cells in a xenograft mouse model. (**A**) Schematic overview of in vivo study. (**B-C**) Kaplan–Meier survival curves of NSG mice treated with PBS, NTD, CD19-CAR, or TET2-1-CD19-CAR T cells one day after injection of Nalm6-Luc cells. The survival rates of mice were monitored up to Day 160 (**B**) or Day 40 (**C**). (**B-C**) Two mice were used per group. (**D**) Bioluminescence image of the mice used in Figure 2(B).
